# Impact of El-Poems study: the e-learning for postural education in music students: a randomized controlled trial protocol

**DOI:** 10.1186/s13063-022-06335-4

**Published:** 2022-05-12

**Authors:** Zahra Akbari-Chehrehbargh, Sedigheh Sadat Tavafian

**Affiliations:** 1 Department of Health Education & Health Promotion, Faculty of Medical Sciences, Tarbiat Modares University, Tehran, Iran; 2 Department of Public Health, Nursing and Midwifery, Faculty of Medical sciences, Karaj Islamic Azad University, Karaj, Iran

**Keywords:** Online Learning, Posture, Music, Students, Musculoskeletal pain

## Abstract

**Background:**

Musculoskeletal pain (MP) has today intensified in a large proportion of music students in Iran. Poor posture while playing an instrument is thus assumed as a very significant risk factor affecting such a condition in this population. The present study aims to evaluate the impact of electronic learning (e-learning) for postural education to music students on posture behavior and MP (abbreviated as an El-Poems study).

**Methods:**

This study is a matched-pair, two-arm, parallel randomized controlled trial (RCT). The participants, as the 7th-to-12th-grade music students, will be accordingly assigned to intervention (*n* = 204) and control (*n* = 204) groups, based on the inclusion and exclusion criteria. The postural education will be also presented through the web-based Student Education Network (with the acronym, SHAD) at the Tehran Conservatory of Music, Tehran, Iran. The intervention program consists of four sessions, using the Integrated Change (I-Change) model. It will be also implemented by a trained physical education instructor and a health educator. The content of the program includes raising awareness, building motivation, and developing skills. Besides, its components are comprised of specific proper postures viz. standing, sitting, lifting, carrying, and hand position while playing a musical instrument. The primary outcome is the MP that will be assessed by the Nordic Body Map (NBM) questionnaire and a numerical rating scale (NRS), and the secondary outcome is the posture behavior that will be evaluated objectively, using the Rapid Entire Body Assessment (REBA) tool. The data will be also collected at baseline and after a six-month follow-up.

**Conclusion:**

This RCT is an innovative study as a pioneer to represent the first attempt for web-based postural education as well as an attractive intervention to prevent MP in Iranian music students.

**Trial registration:**

Current Controlled Trials IRCT20180528039885N2. Prospectively registered on 11 September 2021

## Background

Due to their unique jobs and conditions, music students are at higher risk of playing-related musculoskeletal pain (PRMP), as compared with their peers involved in other disciplines [1–3]. Musculoskeletal pain (MP) in this population is often diagnosed as chronic, and it often occurs because of repetitive movements, poor posture, or carrying heavy musical instruments [1]. However, acute pain is seen due to a sudden increase in instrument practice time [4]. Studies have so far reported the lifetime prevalence of MP in music students by 41–93%, wherein the upper limbs and the spine have been mostly affected [4–8]. In this line, Mehrparvar et al. (2012) had observed that 44.4% of Iranian musicians were suffering from MP [9].

Of note, MP leads to lower quality of life and even significant disabilities in music students, as a serious threat to their performance quality [5, 9–11]. Ultimately, these problems cause depression and anxiety and consequently impose heavy costs on communities. Sometimes, musicians are forced to leave their jobs to recover, but there is an increased likelihood of subsequent injuries, temporary or permanent [12]. Therefore, such problems reveal that a specific postural education program for music students, to guide them how to prevent MP, can significantly improve their performance and quality of life.

Music students at this age are able to assume personal responsibilities for changing their behaviors and choices [13]. Unfortunately, MP prevention behaviors are less common among music students [14, 15], and there is a rising demand for educating them to take care of their own health and adopt proper postures during music practices [14]. According to Salonen (2018), there is a need to develop more prevention programs to promote health and well-being in music students [16]. Receiving education about proper postures and promoting postural behaviors during music practices also play key roles in MP prevention during adulthood and career life [13, 14].

Unfortunately, few studies have thus far implemented and evaluated MP prevention programs among music students [17]. For example, in a recent randomized controlled pilot study by Wolff et al. (2021), the impact of an MP prevention workshop among 57 music students had been assessed. At the 8-week follow-up, the intervention group had accordingly demonstrated a 32% reduction in their pain score, but the controls had shown an 8% increase in pain (*p* < 0.01) [18]. In Davies (2020), the effects of the Alexander Technique classes for music students on MP had been further investigated, and pain reduction had been particularly reported [7]. Cyga´nska et al. (2020) had similarly explored the impact of chair massage and exercise on MP in music students and found an upward trend in pain threshold in both intervention groups compared with the controls [19]. Other studies have also suggested various interventions to relieve MP in music students, using a randomized controlled trial (RCT) [14] and comprehensive health programs during the school year [20]. The educational content in these studies has often covered information about the structure of the musculoskeletal system and the importance of proper posture while playing a musical instrument, wherein the positive effect of such interventions on MP have been confirmed [14, 20]. However, no research has so far developed and implemented an MP prevention program among music students in Iran, to the best of the authors’ knowledge.

In the study by Stanhope (2018), most participants believed that poor posture was a very important risk factor for MP. They generally agreed with the practical workshops about injury prevention strategies, and only 14% of them preferred interactive web-based learning [21]. Before the coronavirus disease 2019 (COVID-19) pandemic [22], education at music conservatories was simply dependent on face-to-face learning, but this disease was a serious threat to this type of learning according to the declaration released by the World Health Organization (WHO) [22].

Although face-to-face learning is a real-time communication between educators and learners, and even provides immediate feedback, it is influenced by place, time, and close contacts [23]. Also, it is alleged that instruction through electronic learning (e-learning) occurs at different times and/or places, from any distance, via various forms of training materials [24–26]. Therefore, the role of e-learning is valuable, particularly for today’s adolescents as digital natives living under pandemic conditions [27].

In this regard, Park et al. (2011) had examined the effect of a web-based program on back health knowledge and self-efficacy among elementary school children [28] and revealed that posture knowledge and self-efficacy had significantly promoted in the intervention groups compared with the controls, but the changes in posture behavior were not significantly different. Also, Ingle et al. (2014) had evaluated the effectiveness of a web-based health promotion course on Playing-Related Musculoskeletal Disorders (PRMDs) in tertiary music students [29], and highlighted the need for postural education to prevent performance-related musculoskeletal injuries. They also believed that this type of research was likely to be applicable to student musicians.

Nowadays, web-based learning or e-learning is extensively used by most students. As a result, it is an effective method to gain access to more audience easily and securely, facilitate wide information sharing, and influence attitudes, beliefs, and health behaviors [30]. Accordingly, this trial aims to evaluate the impact of e-learning for postural education to music students on the prevention and reduction of MP and posture behavior in this group (abbreviated as an El-Poems study).

Among the behavior change models, the Integrated Change (I-Change) one has been so far utilized to explain a variety of health behaviors. It assumes that at least three main phases can be distinguished in the behavioral change process, viz. awareness, motivation, and skills development [15]. Accordingly, raising awareness of MP in music students is the result of their accurate knowledge and risk perceptions about their own posture behaviors. Building motivation to promote posture behavior also depends on music students’ attitudes (i.e., the outcomes of perceived advantages and disadvantages of posture behavior), social influence (namely, norms, posture behavior, and support of others), and self-efficacy expectations (viz. perceived ability to perform posture behavior). Additionally, developing the essential skills for this purpose is required for posture behavior. The three key factors of the I-Change model are thus incorporated into the El-Poems study to provide a scientific explanation for designing health promotion interventions among music students [15].

### Hypotheses

The primary hypothesis is that MP rate and intensity in the intervention group will reduce, compared with the controls. Also, the secondary hypothesis is that the intervention group will modify their posture behavior, compared with the control group.

## Methods

### Trial design

This study is a matched-pair, two-arm, parallel randomized superiority-controlled trial (RCT), which will be conducted at the Tehran Conservatory of Music, Tehran, Iran, in the 2021–2022 school year. The educational intervention is presented through the web-based Student Education Network (with the acronym, SHAD), initiated by the Ministry of Education to pave the way for sustaining the process of education and learning in the country amid the COVID-19 outbreak.

### Participants

The study participants are music students of Tehran Conservatory of Music, Tehran, Iran, in 7th to 12th grades. They will be eligible to enroll in the study if they agree with the conservatory principals and their parents sign a written consent form for their participation. Also, informed consent of voluntary participation in the study, ability to attend the educational sessions, and e-learning experience are among the inclusion criteria. The participants are also excluded from the study if they have previously received postural education, show reluctance for participation, and have a history of underlying diseases, injuries, or disorders or an unhealthy musculoskeletal system.

### The El-Poems intervention

THE intervention program consists of four sessions, each one lasting for 90 min with a 1-week interval. The program will be accordingly implemented by a trained physical education instructor and a health educator. The educational content of the given program is developed with reference to previous studies [11, 12, 14, 15, 18–23]. The music students in the control group also receive a little information about the hearing health. In addition, there are no concomitant interventions that are prohibited during the trial.

The educational program includes three components of raising awareness, building motivation, and developing skills (Table 1), as explained below:

1. *Raising awareness*: It is comprised of a theoretical session to raise awareness and promote knowledge about the importance of proper posture during the playing along with brief explanations about the anatomy and physiology of the musculoskeletal system as well as the basic body posture in relation to playing a musical instrument, warming-up and cooling-down, stretching, relaxation, instrument-specific biomechanics, and ergonomics.
2. *Building motivation*: This component involves one session, including creating positive attitudes toward paying attention to ergonomic postures during playing and benefits of proper posture behavior, social influence (viz. norms, behavior, and support of others), and self-efficacy expectations (namely, perceived ability to perform posture behaviors).
3. *Developing skills*: It consists of two practical sessions, containing attention to body posture during playing a musical instrument, proper posture while standing, sitting, lifting, and carrying instruments, upper limb position, special stretching exercises, and warming-up before playing.

**Table 1:** Content, timeline, components, methods, and responsibilities of the El-Poems study

Content	Components	Methods	Who
Session 1. Promoting in awareness Date: 1–4 December 2021	Improvement of knowledge about importance of proper posture during playing; a brief review of the musculoskeletal system’s anatomy and physiology; to explain about: basic body posture in relation to playing a musical instrument (e.g., standing, sitting, upper limbs, lifting, and carrying position), warming-up and cooling-down, stretching, relaxation, instrument-specific biomechanics, and ergonomics	- Lecture	Main investigator, conservatory health educator
		- Brainstorm, Q & A	
		- Showing Power Point, - Uploading posters, pamphlets, text, ...	
Session 2. Promoting in motivation Date: 6–9 December 2021	Creating a positive attitude toward paying attention to proper postures during playing and its advantages; social influence beliefs (norms of others, behavior of others, and support of others); and self-efficacy expectations (the perceived ability to perform posture behavior)	- Group discussion	Main investigator, conservatory health educator
		- Modeling	
		- Increase risk perception	
		- Benefits of proper posture	
		- Recording participants’ thoughts and experiences about proper posture	
Sessions 3 and 4. Development of skills Date: 11–23 December 2021	Improvement of participants’ abilities to accomplish proper posture behavior: implementation of paying attention to body posture during playing a musical instrument, having proper posture while standing, sitting, lifting and carrying musical instrument, upper limbs position, and practicing special stretching exercise and warming up before playing	- Direct experience	Main investigator, Conservatory physical education instructor and health educator
		- Vicarious experience	
		- Demonstration, re- demonstration	
		- Verbal persuasion	
		- Corrective feedback	

During four online sessions in total, all music students in the intervention group are encouraged to participate in the discussions and ask their questions.

### Outcomes and measures

The participants will complete a short demographic characteristics form containing items about age, gender, grade, instrument type, and weekly playing time. The primary outcomes are:

1. The presence of MP, which is assessed using the Nordic Body Map (NBM) questionnaire
2. The intensity of pain that will be evaluated via a numerical rating scale (NRS) questionnaire, wherein the participants are asked to circle the number between 0 and 10 that fits best to their pain intensity. Also, zero represents “no pain at all” whereas pain ratings between 1 and 3 indicate mild pain, 4 to 6 show moderate pain, and 7 to 10 denote severe pain

To collect these self-reported data, a link to the online questionnaire is posted in two WhatsApp groups supervised by the class teacher. In addition, there are no missing data due to the obligation to answer all questions.

**Fig. 1:**
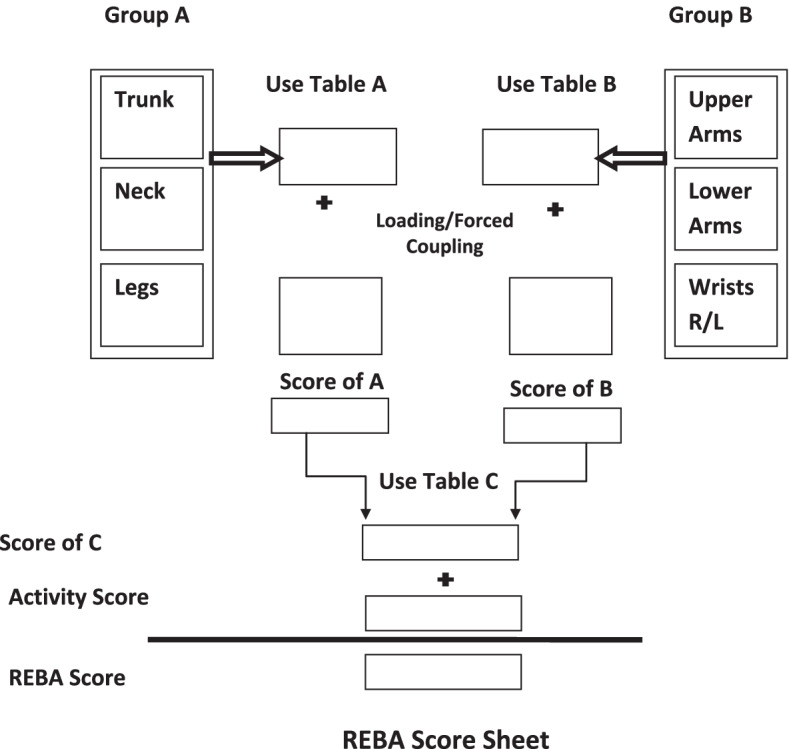
Rapid Entire Body Assessment (REBA) score sheet (Ahmadi et al. 2015)

The secondary outcome takes account of posture behavior, estimated through the Rapid Entire Body Assessment (REBA) tool by observing and filming the body positions of the music students during specific postures while playing a musical instrument through the SHAD platform. One independent trained rater blinded to the study will also observe and record the participants’ postures. Each posture is initially given a score. Then, the posture scores are estimated based on the diagram shown in Fig. 1. The final REBA score will be between 1 and 15 and divided into five levels in terms of risks: 1 = negligible risk, 2–3 = low risk, 4–7 = moderate risk, 8–10 = high risk, and 11–15 = very high risk [31].

The participants’ data are collected at two points in time, namely, at baseline (1 week before the educational program) and 6 months after it. To prevent attrition and bias, the data are collected from all participants and included in the data analysis, regardless of adherence to the study protocol. The participants will also receive a small gift for their participation.

### Sample size

Sample size calculation is based on the study by Mehrparvar et al. (2012) on MP among Iranian instrumentalists [9], wherein the prevalence of MP in music students had been reported about 44% (p1 = 0.44). If it is assumed that the given rate will decrease to 30% (p2 = 0.30) after the El-Poems intervention, a trial consisting of 185 music students per group would have 80% power (*β* = 0.2) at the 5% significance level (*α* = 0.05). To assume a 10% dropout for each group, at least 204 samples in each group will be needed for the El-Poems study. The following formula is accordingly applied to calculate the sample size:

$$n=\left\{{\left({Z}_{1-\upalpha /2}+{Z}_{1-\upbeta}\right)}^2\times \left[{p}_1\left(1-{p}_1\right)+{p}_2\left(1-{p}_2\right)\right]\right\}/{\left({p}_1-{p}_2\right)}^2$$

### Randomization

**Fig. 2:**
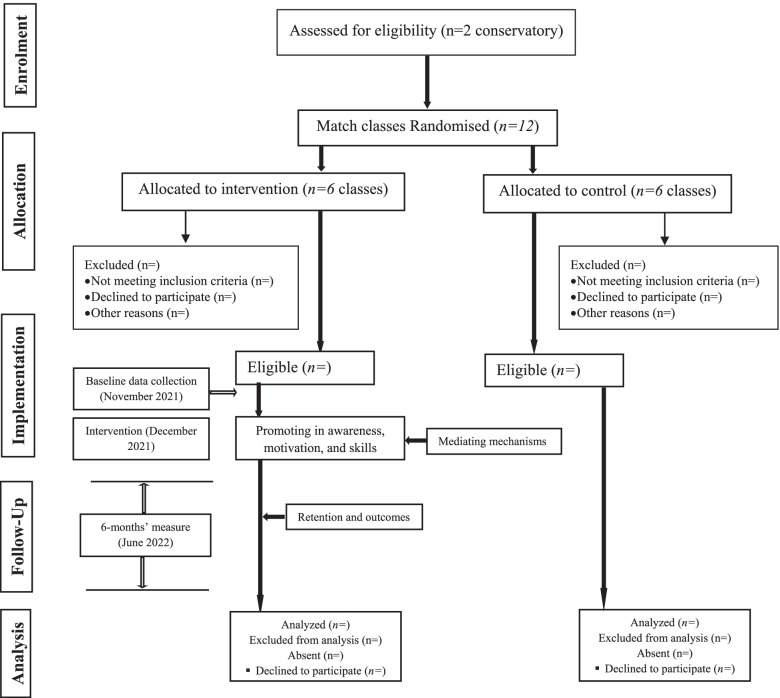
The El-Poems study process flow diagram

Totally, there are two national music conservatories in Tehran, Iran. Since individual allocation is not possible in a conservatory’s context, the classes at each conservatory are first randomly divided into two matched groups based on the number of students, grade, and gender. Then, the groups are numbered as 1 and 2 and placed in the bowl. A neutral person is next asked to pull out one of the numbers from the bowl. The group number 1 is thus assigned to the intervention group and 2 is given to the controls. The main researcher also supervises the randomization steps. The current RCT protocol is reported in compliance with the Consolidated Standards of Reporting Trials (CONSORT) guidelines. The El-Poems study flowchart is depicted in Fig. 2.

### Statistical analysis

The data will be analyzed using the SPSS Statistics software package 24.0 (IBM SPSS 24.0 Ink, NY: IBM Corp). All continuous variables will be also assessed for their normal distribution. *T*-test, Mann-Whitney *U* test, and chi-square test will be then utilized to compare the baseline measurements. The descriptive statistics are also reported to investigate the data. To obtain the time period (at baseline and 6-month evaluation, as the test time factor) in which the difference in mean scores within the groups (viz. group factor) originates, the two-way analysis of variance (ANOVA) will be exploited. The test for homogeneity of the data is further employed for the between-participant variables. Since we do not have a variable with more than one pair, confidence interval adjustment is not selected. The Wilcoxon signed-rank test is additionally calculated for the difference in the MP rate between both study groups. Moreover, the effect sizes will be calculated using the partial eta-squared measure (ηp^2^) that gives the idea of how different the participants are. The significance level will be set at *p* < 0.05.

### Timeline

Table 1 shows the timeline for each session and task in the intervention, as well as who performs each task.

## Discussion

The need for prevention programs for the health and well-being of music students is well-documented [16]. However, little information is usually presented to music students about the impact of PRMP. Although a conservatory seems an ideal context to support the improvement of health of a considerable population, only few studies have so far assessed the influence of prevention programs on music students’ health and well-being [17], to the best of the authors’ knowledge. This RCT is the first attempt in Iran to evaluate the impact of an e-learning postural education on MP among music students.

It is hypothesized that the El-Poems intervention promotes posture behavior and raises effective prevention of MP in the intervention group compared with the controls. The study results are also assumed to inform the future implementation of this El-Poems study in other music education settings more broadly. Moreover, it is a good idea to share the RCT protocol to both music teachers and health educators, who may want to use it in their music education or interventions.

Therefore, this RCT is an innovative study as a pioneer to represent the first attempt of web-based postural education as well as an attractive intervention to prevent MP among Iranian music students. The theoretical framework for posture behavioral change is to raise awareness, build motivation, and develop skills. In order to improve the proposed key factors, effective methods will be also applied as with the previous study [32] (Table 1). This intervention may be delivered via face-to-face learning.

An advantage of this trial is using an observational tool to measure posture behavior and a standard questionnaire to assess MP. The other strength of this study is the implementation of a matched-pair, two-arm parallel RCT in a relatively large sample, including both female and male music students and all grades at a conservatory. Finally, the El-Poems study has the potential to make MP prevention programs widely accessible and reduce the current costs in educational centers.

The study limitations are also related to the nature of e-learning interventions. In the face-to-face learning, educators can control the instruction dosage by leading learners through training sessions. E-learning interventions, however, do not allow educators to transmit the content straightly, so they have to control the dosage. In the El-Poems study, there were attempts to address this limitation by asking the participants about the content and monitoring the amount of their attention to the program through the question & answer method (Q&A). Secondly, the lack of group discussion will be another limitation, associated with the nature of e-learning interventions. The group discussion is a very important method for the success of such interventions [33]. However, in the El-Poems study, opportunities for discussion, reflection, and participant engagement with the educators will be considered, where the participants are requested to share their questions, thoughts, and experiences about proper posture, MP, and the intervention.

## Conclusion

It is concluded that music conservatories in Iran need to receive prevention programs in order to enhance proper posture behavior and reduce MP among music students. Therefore, interventions that can target posture behavior at conservatories, with enough feasibility in the long run, should be implemented. The El-Poems study can be thus an available health promotion intervention, particularly during the COVID-19 pandemic.

## Trial status

IRCT registration number: IRCT20180528039885N2

Registration date: September 11, 2021

Last update: September 11, 2021

Update count: 0

Country: Iran (Islamic Republic of)

Name of organization/entity: Tarbiat Modares University

Contact for public queries: ZAC, PhD, z-akbari@modares.ac.ir

Contact for scientific queries: SST, PhD, No 212, Department of Health Education & Health

Promotion, Faculty of Medical Sciences, Tarbiat Modares University

Registration timing: prospective

Recruitment status: Not yet recruiting

Expected recruitment start date: December 1, 2021

Expected recruitment end date: May 31, 2022

Trial Sponsor: Iran National Science Foundation

Contact name: Iman Eftekhari

Address: No. 33, 5th St., North Karegar Ave., Tehran, Iran

Telephone: +98 21 8216 1000

Email: info@insf.org

## Data Availability

At this time, the data is not publicly available because the work is continuing. However, the final dataset will be available from the corresponding author on reasonable request. The study results will be released to the participants, Ministry of Education and District, healthcare professionals, the public, and other relevant groups.

## Abbreviations

El-Poems: E-Learning for postural education in music students
I-Change: Integrated change
η_p_^2^: Partial eta squared
Q&A: Question and answer
RCT: Randomized controlled trial
REBA: Rapid Entire Body Assessment
